# ‘Total pain’: reverence and reconsideration

**DOI:** 10.3389/fsoc.2023.1286208

**Published:** 2023-11-23

**Authors:** Maxxine Rattner

**Affiliations:** Lyle S. Hallman Faculty of Social Work, Wilfrid Laurier University, Kitchener, ON, Canada

**Keywords:** total pain, suffering, palliative, hospice, psychosocial, existential

## Abstract

Dame Cicely Saunders’ conceptualization of ‘total pain’, or ‘total suffering’, is one of her most significant and lasting contributions to the field of palliative care. It was Saunders’ unique combination of knowledge and experiences as a trained social worker, nurse and physician that influenced her understanding of suffering specific to a life-limiting illness as being multi-dimensional: that suffering may be simultaneously physical, psychological, emotional, social, spiritual and/or existential in nature. ‘Total pain’ remains a highly relevant and significant concept within palliative care and Saunders’ lasting contributions are to be revered. This paper invites us to reconsider one particular aspect of Saunders’ conceptualization: that patients’ ‘mental reactions’ to their anticipated dying/death is a key contributor to their ‘total pain’. Drawing upon Saunders’ works from the late 1950s to the early 2000s, this paper details the socio-historical manifestation of this aspect of ‘total pain’ within Saunders’ writings, including influences from her Christian religion and Viktor Frankl, and its enduring impact on palliative care philosophy, practice, and discourse. Then, drawing upon patient stories rooted in my own clinical experiences over a 10 year period as a hospice social worker, I suggest that this particular feature of Saunders’ ‘total pain’ may, unintentionally, work to pathologize both the patient for whom suffering persists and remains unsolvable, and the palliative care clinician who may struggle to relieve it — and why it therefore stands to be revisited. It is my sincere hope and intention that ongoing reverence for Saunders’ significant contributions can sit alongside respectful reconsideration.

## Introduction

Palliative care has come to know and understand ‘suffering’ from Dame Cicely Saunders’ conceptualization of ‘total pain,’ also referred to at times as ‘total suffering.’ Saunders’ conceptualization of ‘total pain’ importantly denotes the multidimensional aspects of suffering specific to a life-limiting illness: that suffering may be simultaneously physical, psychological, emotional, social, spiritual and/or existential in nature (Clark, 2016). It was Saunders’ unique combination of knowledge and experience as a trained social worker, nurse and physician that influenced how she came to know and understand ‘total pain’ as comprising both physical *and* non-physical suffering — a revolutionary idea at the time, in the late 1950s/early 1960s, when solely physical pain and suffering was the focus in medical care (Krawczyk and Richards, 2018). As a foundational clinical concept in hospice and palliative care, ‘total pain’ continues to shine an essential light on how physical and non-physical suffering can, at times, be complexly intertwined: for example, how physical pain may be amplified by, and difficult to relieve because of non-physical sources of suffering, or how “if physical symptoms are alleviated then mental pain is often lifted also” (Saunders, 1963, p. 746). For these reasons, and many others, ‘total pain’ remains a highly relevant and much needed concept in palliative care — and beyond. Saunders’ role in coining this foundational concept and shaping how palliative care has come to know and understand suffering, is to be revered.

At the same time, there is one aspect of Saunders’ conceptualization of ‘total pain’ that stands to be respectfully revisited and reconsidered. While Saunders is said to have first elaborated on her conceptualization of ‘total pain’ in 1964 (Krawczyk and Richards, 2018), it was 5 years earlier when she wrote the following in *Care of the dying 3. Control of pain in terminal cancer*: “Much of our total pain experience is composed of our mental reaction...” (Saunders, 1959, p. 1082). This is an idea that would permeate her writings for decades to follow, such as in the following excerpt from the introduction of her co-edited book, *The management of terminal malignant disease* in 1993: “If we are held in suffering, we then have responsibility for the attitude in which we suffer” (Saunders, 1993, p. 10). For Saunders, then, how a patient mentally responds to their life-limiting diagnosis and anticipated dying/death contributes to whether and/or how they suffer or experience ‘total pain’.

In this paper, I will first detail the socio-historical manifestation of this particular aspect of ‘total pain’ in Saunders’ writings, including influences from Viktor Frankl and her Christian religion, and its lasting impact within the field of palliative care. Then, drawing upon patient stories rooted in my own clinical experiences over a 10 year period, I will suggest that this particular feature of Saunders’ ‘total pain’ may, unintentionally, work to pathologize both the patient for whom suffering persists and remains unsolvable, and the clinician who may struggle to relieve it — and why it therefore stands to be revisited. It is my hope and intention that ongoing reverence for Saunders’ significant contributions can sit alongside respectful reconsideration.

## Socio-historical overview

I begin by providing a socio-historical overview of Saunders’ writings that detail how she came to think that patients’ own “mental reaction[s]” to their life-limiting illness and anticipated dying/death is a key contributor to their ‘total pain’ (Saunders, 1959, p. 1082). Specifically, I will highlight how the works of Viktor Frankl and her devout Christianity shaped her thinking.

### Influence: Viktor Frankl

Viktor Frankl’s famous book, *Man’s Search for Meaning*, was first published in 1959, a formative time in Saunders’ visioning. Saunders is known to have first encountered Frankl’s work in 1963 (Clark, 2016); his writings would directly influence her thinking about suffering. Frankl’s (1959) ideas about suffering were formed when he was imprisoned in a Nazi concentration camp: “Suffering ceases to be suffering at the moment it finds a meaning” (p. 113), and “If, on the other hand, one cannot change a situation that causes his suffering, he can still *choose his attitude*” (p. 148, emphasis added). These statements strike at the very core of Frankl’s beliefs: meaning can bring suffering to an end; and amid suffering, we have the ability to choose how we respond to it. While his book focuses mainly on his time imprisoned in the camp, and his efforts to bolster the spirits of his fellow prisoners amidst the horrors of camp life, Frankl (1959) extrapolates from that context to one of an individual with an incurable illness, when he writes,

Take the fate of the sick—especially who are incurable. I once read a letter written by a young invalid, in which he told a friend that he had just found out he would not live for long, that even an operation would be of no help. He wrote further that he remembered a film he had seen in which a man was portrayed who waited for death in a courageous and dignified way. The boy had thought it a great accomplishment to meet death so well. Now—he wrote—fate was offering him a similar chance (p. 68).

For Frankl, then, individuals with a life-limiting illness can accomplish much by choosing to meet — or ‘mentally respond’ to — their anticipated death with courage and dignity. Frankl (1959) applies his idea of meaning to suffering and dying from a life-limiting illness, when he writes: “In accepting this challenge to suffer bravely, life has a meaning up to the last moment, and it retains this meaning literally to the end” (p. 114). Saunders would soon write about the utility of Frankl’s thinking to her own work; in a 1966 article titled, *The care of the dying*, she notes: “We can learn from Frankl, a psychiatrist, who found a purpose and meaning in life and death in Auschwitz” (p. 141). In the same text, she writes: “If we are held in suffering, we then have responsibility for the attitude in which we suffer” (Saunders, 1993, p. 10) — a nearly direct quote from Frankl himself. Inspired by Frankl, Saunders’ writings embodied the idea that one’s attitude, one’s mental reaction, shapes one’s suffering.

### Influence: Christianity

While Saunders’ views on suffering were greatly influenced by Frankl (Clark, 2018), they were also keenly informed by her devout — and at times evangelical — Christian background (Clark, 2018; Krawczyk et al., 2018). In his book, *To comfort always: A history of palliative medicine since the nineteenth century*, Clark (2016) writes that Saunders drew, “…on a wide range of clinical, *religious*, and cultural influences to formulate her particular approach to the care of the dying” (p. 86, emphasis added). Saunders described St. Christopher’s, the hospice she founded in 1967 in London, England, as follows: “St. Christopher’s Hospice is defined as a religious foundation based on the full Christian faith in God” (Clark, 2016, p. 91). While Christianity was not imposed on St. Christopher’s patients (Saunders, 1966, 2001), religious ideas would underlie and influence her approach to suffering and its relief (Krawczyk et al., 2018). The interplay between Saunders’ Christian beliefs and her belief that one’s own mental reaction determines one’s suffering can be seen in the following text from a book she co-wrote in 1995 titled, *Living with dying: A guide to palliative care:*

A feeling of meaninglessness that neither oneself nor the universe itself has permanence or purpose, is a form of spiritual pain. Patients need to look back over the story of their lives and believe that there was some sense in them and also to reach out towards something greater than themselves, a truth to which they can be committed. This is often linked with the belief that somehow life goes on…the belief (or perhaps it would often be better described as a feeling or intuition) that our visible, physical life is not the whole of our personal history is exceptionally tenacious…that ‘God can and will re-create our being even beyond annihilation’, looked beyond to another dimension where the individual’s capacity to love and worship will be fulfilled in freedom (Saunders et al., 1995, p. 55).

In this passage, Saunders shares the idea that, at the end of life, reflecting on and making sense of one’s life, as well as believing in an afterlife, can help to create meaning to assuage one’s “spiritual pain” or suffering. A 1983 book written by Saunders, *Beyond all pain: Companion for the suffering*, provides another example of how her religion, together with Frankl’s ideas, shaped her thinking and practice. She describes the book at its start in this way, “This book includes contributions written or dictated by patients in St. Christopher’s Hospice who searched for meaning as they faced progressive illness and disability. What they wrote has helped many who knew them” (Saunders, 1983, p. viii). The majority of the book’s readings are of a religious nature, with section titles such as, “Search for meaning,” “Meaning,” “Suffering,” “Dying,” and “Resurrection”. These are shared as just a few examples of how Saunders’ Christian ideas, in addition to Frankl’s, are foundational to her thinking about suffering.

Linked with her interest in meaning-making as an antidote to suffering, and connected to her underlying Christian orientation, Saunders also consistently wrote about dying being a time that is imbued with acceptance, personal growth and reconciliation — additional, and particular, ‘mental reactions’ to one’s dying. The achievement of “serenity” — feeling peaceful, and untroubled — is described as an aim and accomplishment in Saunders’ early writings. In a 1959 article, Saunders describes a 54 year old woman, Mrs. W, who entered the hospital “…frightened of hospital and of death and was very tense and tearful on arrival.” (p. 1031). That Mrs. W was able to find “peace and acceptance never failed to the end and compelled admiration from all of us” (Saunders, 1959, p. 1031) — here, the “us” refers to Saunders and the clinicians caring for Mrs. W. In 1963, in a letter published in the *British Medical Journal* titled *Distress in Dying*, Saunders writes:

It is often not realized how much can be done to enable these patients, and their families also, to make an achievement of this part of their lives as of any other. At St. Joseph’s Hospice we do not see intractable fear and depression but rather growth and acceptance and serenity (Saunders, 1963, p. 746).

Here, the idea that clinicians can enable patients and their families to make end of life a time of achievement is presented, as is the idea that the end of one’s life should be mentally responded to in particular ways — as an opportunity for growth, acceptance and serenity. In another article written in 1964, Saunders captions a photo of a smiling patient with, “This patient died only four days later. We will not forget her achievement of serenity although she was only with us for a short time” (Saunders, 1964, p. x). And referencing the end of one’s life, Saunders would write decades later that, “Surprising growth can be achieved in a short time, as in all situations of crisis” (Saunders et al., 1995, p. 45).

Related to fostering personal growth, Saunders also calls for clinicians to facilitate patients’ “reconciliation” and “fulfillment” at end of life. She wrote the following, referring to the work of palliative care clinicians:

Our work is to try to alter the character of this stage of illness not only so that such distress should be relieved but also that this time is not seen or thought of as a long defeat but as a positive achievement in dying itself, a time for reconciliation and fulfillment for the patient and perhaps his family also (Saunders, 1966, p. 137).

That patients should respond to their dying ‘positively’ is also captured in a 1968 paper in a Catholic quarterly; “it called for a positive approach that sees this as a time not of defeat but of life’s fulfilment, recognizing that there will be many different paths to life’s ending” (Clark, 2016, p. 104). Nearly 30 years later, Saunders echoed these ideas, noting that at end of life, “…reconciliation is not uncommon, and many people make this a remarkably fruitful time” (Saunders et al., 1995, p. 50).

## Enduring impact on palliative care

Saunders drew upon the works of Viktor Frankl and her devout Christian background when she espoused the ideas that meaning is the antidote to suffering (Saunders, 1966), and that patients can choose to respond to their suffering by finding peace, serenity, and meaning, reaching ‘acceptance,’ being ‘positive’ and experiencing personal growth (Saunders, 1959, 1964, 1966). Saunders’ writings provide insight into her thoughts on how a patient ideally mentally responds to their anticipated dying and death, and how she believed that one’s mental reaction can work to mitigate one’s own suffering, or ‘total pain’. That clinicians have a role in facilitating these ends for patients, even admiring patients who are able to mentally respond in these particular ways, is also visible in Saunders’ writings (Saunders, 1959, 1964, 1966). These understandings of suffering continue to widely influence palliative care philosophy, practice and discourse more than 50 years later: whenever suffering is written about within palliative care, ideas around meaning-making, personal growth, and interventions to help alter how a patient thinks about — or mentally responds to — their situation, abound. So, too, does the idea that it is the role of the palliative care clinician to help patients achieve these aims (Rattner and Berzoff, 2016; Rattner, 2019, 2022, 2023).

## Reverence and reconsideration

I have written before about the potentially pathologizing nature of meaning-making, personal growth, finding peace, and acceptance as antidotes to suffering (Rattner and Berzoff, 2016; Rattner, 2019), at the time not knowing the socio-historical roots of these ideas. My doctoral work allowed time and space for the archaeological exploration I detail in this paper (Foucault, 1972). Understanding how a field ‘comes to know’ something is important, as how something is ‘known’ and framed largely determines what is done in response. Palliative care has — both gratefully and understandably — come to know and understand ‘total pain’ and suffering from Saunders’ early conceptualizations. While these ideas and approaches can and do deeply resonate with and support many patients in their suffering, in my experience, they do not capture the whole of how patients respond to their suffering, or what it can be like for clinicians to encounter patients in their suffering and ‘total pain’ that persists. A great many, if not most, of the patients I encountered in my 10 years of bedside hospice practice struggled to mentally respond to their dying in the ways espoused by Saunders. Many, if not most, struggled to find meaning in their dying, to be ‘positive,’ to reach a place of ‘acceptance,’ or to use the ‘dying time,’ as I have come to call it (Rattner, 2023, p. 120), as an opportunity for personal growth. For many, dying was unchangeably hard, imbued with losses, worries and fears that a recent scoping study revealed are entirely common to living with, and dying from, a life-limiting illness across diverse cultures and geographies (Rattner, 2022). If patients I worked with did experience moments of meaning, personal growth, or acceptance, I found that such moments commonly sat alongside all of the difficult emotional and practical realities of leaving one’s life and one’s people; they sat alongside, and not in the absence of, suffering (Rattner and Berzoff, 2016).

Perhaps it is a patient’s worry about how family members will cope after they die that is contributing to their ‘total pain.’ In instances like this, family members, including chosen family, can try to reassure the patient they will be okay after they die, and even note the various support services that may be available that they intend to access; but I have found more often than not, the patient’s worry itself — their suffering — does not go away. Another example is when a patient does not want to die, another salient source of ‘total pain,’ and one that is seldom named in palliative care’s discourse (Rattner, 2023). If a patient’s ‘not wanting to die’ is related to a fear they have of the dying process, a palliative care team can sensitively explain the dying process and what to expect as they move closer to dying; this can be, though is not always, a source of comfort to the listening patient. If they are worried or fearful about the moment of death or what comes after, we may explore with them what ideas about death and what comes after may bring them comfort, if there are any such ideas for them. I have encountered patients of all spiritual and religious backgrounds who found no such comfort; this is a source of suffering that is difficult to assuage. But ‘not wanting to die’ can also be very different from death anxiety and/or existential distress; it can be an enduring aspect of profound grief that, in my experience, regularly contributes to ‘total pain.’ And if ‘not wanting to die’ is about not wanting to leave one’s life and one’s people behind, this is another aspect of a patient’s ‘total pain’ experience that is entirely difficult, if not impossible, for clinicians to quell. In such moments, I have witnessed patients rebuke the idea that reflecting on a meaningful life is meant to help them in such moments of profound grief. I would regularly encounter patients who had lived very meaningful lives who were struggling to find meaning in, accept, or experience personal growth in their dying and anticipated death. I would also consistently encounter patients for whom reflecting on their life and their accomplishments did not relieve their fear of dying, or death, or what happens after we die — additional, common contributors to ‘total pain.’ In my clinical experience, meaning does not necessarily shield one from suffering.

Can the idea that patients can ease their own suffering by mentally responding to their suffering in a particular way — by finding meaning, reaching acceptance, peace and serenity and experiencing personal growth — work to unintentionally pathologize the patient for whom suffering persists? Might it work to create expectations within patients, their families, and clinicians for patients to cope in these particular — seemingly preferable — ways? Many patients I worked with would regularly express feeling that they should be doing their dying differently than they were, and this internalized expectation was itself a source of suffering. As one example, I recall a patient in his 70’s — a dad, husband, and grandfather — who experienced complex ‘total pain.’ During one bedside encounter, he shared with me that he felt like he was not doing his dying ‘right,’ that he was trying to draw on coping mechanisms that had always helped him in the past — like “being positive” (his words) — but was finding that this was not working. When I shared with him that dying was ‘new territory’ as he had never done it before (as I had learned from other patients I had worked with), and that it therefore made sense that past coping mechanisms were not working as they always had, he shared feeling a great relief — not relief from his suffering, but of the pressure to feel differently than he did. He was relieved to know that dying an anticipated death is, perhaps, different from other challenges he had faced in his life, and that it was okay that he was having a hard time. He felt reassured knowing that the expectations he had placed on himself were not really fair.

I think, too, of a young mom who I once worked with — her ‘total pain’ looked like this: holding both ‘wanting to die’ a natural death and ‘not wanting to die’ in equal measure, alongside complex physical symptoms — another example of suffering that is not readily acknowledged in the palliative care literature and discourse. She both wanted to live to spend as much time as possible with her young children, but she also did not want her dying to take too long because her quality of life had diminished so significantly due to the physical burden of her illness; an example, perhaps, of “self conflict,” a particularly difficult feature of suffering (Cassell, 2004, p. 274) and key contributor, in my clinical experience, to ‘total pain.’ Legacy work did not relieve her suffering, and she did not find meaning, peace or acceptance in her dying, nor did she experience personal growth — it was, understandably, a deeply difficult time for her and her family.

Elsewhere, I wrote about a patient who struggled to feel ‘accepting’ of her dying; specifically, it was,

…. a mother in her 60’s, [who] said to me, “Maxxine, can I die without accepting that I am dying?” She of course knew within herself that she was going to die, but she did not like it; for her, accepting death – another ideal way of coping upheld within palliative care’s discourse – felt like a betrayal to the life that she loved and wanted to keep living, despite knowing that her time was limited (Rattner, 2019).

When I reassured her that she did not have to be ‘accepting’ of her dying, and that she could hold all of the feelings that came up for her, without needing to alter her mental and emotional response, she was relieved — not of suffering, but of the pressure to feel differently than she did (Rattner, 2019). I came to spend much of my time as a hospice social worker doing this very thing — validating and normalizing patients’ experiences of suffering, and assuring them that they did not need to feel — or mentally respond — any differently than they did (Rattner and Berzoff, 2016). And for them, *this* was a source of relief.

Patient stories like these are what move me to suggest that Saunders’ (1959) idea that patients’ own “mental reaction[s]” (p. 1082) to their life-limiting illness and anticipated dying/death is a key contributor to their ‘total pain’ stands to be respectfully revisited. Yes, some patients will be able to shift or “alter” (Saunders, 1966, p. 137) how they mentally react to their life-limiting diagnosis, anticipated dying and/or death; some, will not. Some will find meaning in, accept, feel peaceful about, and experience personal growth in their dying; many will not. More likely, many will oscillate between these and a variety of ‘mental reactions’ and ways of coping within the same day, hour, or even moment. For some, *not* changing their mental reaction and being allowed to feel however they feel is what might offer great reprieve. Each of these possibilities needs to be understood, validated, and supported within the delivery and practice of palliative care. So, too, does the idea that changing one’s ‘mental reaction’ to one’s anticipated dying/death — for reasons like the ones outlined above — may be outside the purview of a patient’s, or clinician’s, control. Alongside reverence for Saunders’ remarkable contributions, palliative care’s collective understanding of ‘total pain’ and suffering stands to benefit from this seemingly small, yet potentially significant, reconsideration.

## Data availability statement

The original contributions presented in the article are included in the article/Supplementary material; further inquiries can be directed to the corresponding author.

## Author contributions

MR: Writing – original draft.
